# Phosphorylation of adducin-1 by TPX2 promotes interpolar microtubule homeostasis and precise chromosome segregation in mouse oocytes

**DOI:** 10.1186/s13578-022-00943-y

**Published:** 2022-12-20

**Authors:** Ying Zhang, Bingfeng Fan, Xiaoxia Li, Yu Tang, Jing Shao, Lixiang Liu, Yuhe Ren, Yifeng Yang, Baozeng Xu

**Affiliations:** 1 Institute of Special Animal and Plant Sciences, Chinese Academy of Agricultural Sciences, #4899 Juye Street, Jingyue District, Changchun, 130112 Jilin China; 2 State Key Laboratory for Molecular Biology of Special Economic Animals, Institute of Special Animal and Plant Sciences, Chinese Academy of Agricultural Sciences, Changchun, Jilin China; 3 College of Animal Science and Technology, Jilin Agriculture Science and Technology University, Jilin, China

**Keywords:** Mouse oocyte, Interpolar microtubule stability, Acentriolar spindle assembly, Aneuploidy, ADD1, TPX2

## Abstract

**Background:**

ADD1 (adducin-1) and TPX2 (targeting protein for Xklp2) are centrosomal proteins and regulate mitotic spindle assembly. Mammalian oocytes that segregate homologous chromosomes in Meiosis I and sister chromatids in Meiosis II with a spindle lacking centrosomes are more prone to chromosome segregation errors than in mitosis. However, the regulatory mechanisms of oocyte spindle assembly and the functions of ADD1 and TPX2 in this process remain elusive.

**Result:**

We found that the expression levels and localization of ADD1, S726 phosphorylated ADD1 (p-ADD1), and TPX2 proteins exhibited spindle assembly-dependent dynamic changes during mouse oocyte meiosis. Taxol treatment, which stabilizes the microtubule polymer and protects it from disassembly, made the signals of ADD1, p-ADD1, and TPX2 present in the microtubule organizing centers of small asters and spindles. Knockdown of approximately 60% of ADD1 protein levels destabilized interpolar microtubules in the meiotic spindle, resulting in aberrant chromosome alignment, reduced first polar body extrusion, and increased aneuploidy in metaphase II oocytes, but did not affect K-fiber homeostasis and the expression and localization of TPX2. Strikingly, TPX2 deficiency caused increased protein content of ADD1, but decreased expression and detachment of p-ADD1 from the spindle, thereby arresting mouse oocytes at the metaphase I stage with collapsed spindles.

**Conclusion:**

Phosphorylation of ADD1 at S726 by TPX2 mediates acentriolar spindle assembly and precise chromosome segregation in mouse oocytes.

**Supplementary Information:**

The online version contains supplementary material available at 10.1186/s13578-022-00943-y.

## Background

Mammalian oocytes initiate meiosis during fetal life and are arrested in prophase I until reproductive maturity in mammals. Stimulated by the luteinizing hormone, the fully grown oocytes resume meiosis and complete meiosis upon fertilization. Meiosis of oocytes divides their chromosomes equally and asymmetrically segregates the cytoplasm to produce a large haploid egg, which allows most of the maternal components to remain in the egg for fertilization and early embryonic development. It has been known that meiosis of oocytes is more prone to chromosome segregation errors than meiosis in males and mitosis in somatic cells [1–3]. These segregation errors frequently cause aneuploidy, which results in the loss of the conceptus during pregnancy or birth defects [4, 5]. The meiotic spindle is the cellular apparatus essential for the proper alignment and segregation of chromosomes to maintain euploidy during meiotic cell division in gametes [6, 7]. Therefore, any abnormity in meiotic spindle assembly or its association with chromosomes could cause aneuploidy in oocytes. Unlike somatic cells, mammalian oocytes lack centrosomes that serve as major sites of microtubule nucleation and form the two poles of the mitotic spindle in somatic cells [1–3, 8–10]. Furthermore, the meiotic spindle in mammalian oocytes is devoid of astral microtubules that emanate from the centrosome and regulate the mitotic spindle translocation through physical interaction with the cell cortex in somatic cells [1–3, 11, 12]. Meiotic spindle assembly is further complicated by the unique requirement that the sister chromatid kinetochores must be stuck on microtubules from the same spindle pole (mono-orientation) during Meiosis I, whereas the kinetochores of sister chromatid are attached to microtubules emanating from opposite poles (bi-orientation) during Meiosis II. A comprehensive understanding of the mechanisms by which mammalian oocytes assemble the meiotic spindle and regulate chromosome alignment/segregation remains a fundamental question.

Microtubule nucleation is a prerequisite for spindle assembly. Mechanistic details about microtubule nucleation pathways and their coordination are beginning to be revealed. Canonical centrosomes are composed of a pair of centrioles surrounded by pericentriolar materials (PCMs) that possess microtubule nucleation activity. Mouse oocytes lack centrioles but contain some PCMs, such as centrosomal proteins 120 and 192 (CEP120, CEP192), γ-tubulin, pericentrin, NuMa, and NEDD1, which are termed as acentriolar microtubule organizing centers (aMTOCs) and function as main centers of microtubule nucleation in meiosis [1–3, 13–19]. One discrepancy between Meiosis I and Meiosis II is that the chromatin-dependent Ran-GTP pathway is essential for spindle formation in Meiosis II but not for Meiosis I in mouse and X. laevis oocytes. However, Ran-GTP did enhance microtubule nucleation from aMTOCs, allowing rapid spindle assembly during Meiosis I in both modles [18]. The disruption of aMTOCs function in mice with oocyte-conditional knockout of pericentrin, an essential aMTOC protein, causes meiotic spindle instability and severe meiotic errors that lead to pronounced female subfertility [19]. These defects demonstrate that aMTOCs-driven spindle assembly is dominant over the aMTOCs-independent pathways during Meiosis I of mouse oocytes, including the Ran-GTP pathway. In contrast, human oocytes assemble meiotic spindles through the chromatin-dependent Ran-GTP pathway rather than aMTOCs, which lack pericentrin and γ-tubulin [20]. In addition, microtubule-dependent microtubule generation, catalyzed by the eight-subunit protein complex of Augmin, provides another pathway that contributes to overall spindle microtubule density and spindle bipolarization [21, 22]. Although it has been shown that different cells use different molecular pathways for microtubule nucleation to generate bipolar spindles, little is known about how these pathways are coordinated within a single cell.

TPX2 (targeting protein for Xklp2) is a multifunctional microtubule-associated protein that regulates spindle assembly and function in the chromatin-dependent Ran-GTP and Augmin-dependent microtubule nucleation pathway [23–25]. Inhibition of Ran-GTP delays but does not impair the assembly of the functional spindle of Meiosis I in mouse and X. laevis oocytes [18]. In contrast, TPX2 depletion triggers severe perturbation in Meiosis I spindle assembly with only a few microtubules present around the chromosomes in most of the investigated mouse oocytes [26]. These discrepancies suggest that TPX2 may play important biological functions in other microtubule nucleation pathways besides the Ran-GTP-dependent microtubule nucleation pathway. Evidence shows that TPX2 can directly recruit γ-TuRC as well as Augmin, which in turn targets more γ-TuRC along the microtubule lattice to efficiently nucleate microtubules from preexisting microtubules [27]. Although an increasing number of studies have demonstrated that TPX2 is required for meiotic spindle assembly via regulating microtubule assembly and spindle pole integrity [20, 26], how exactly TPX2 mediates the meiotic spindle assembly in mammalian oocytes remains unclear.

Adducin-1 (ADD1) has been shown to play important roles in the stabilization of the membrane cortical cytoskeleton [28], cell–cell adhesions[29], and spindle assembly [30–32]. S726-phosphorylated ADD1 localizes to centrosomes, where it interacts with TPX2 and organizes into a rosette-like structure at the pericentriolar material in mitotic cells [31]. ADD1 knockdown results in distorted, elongated, and multipolar spindles with aberrant chromosomal alignment [30], as well as centriole splitting and defective spindle pole integrity [31] during mitosis. This multipolar spindle defect can be rescued by the phosphomimetic ADD1 S726D mutant, but not by the dephosphomimetic ADD1 S726A mutant [31], which restores the distortion and elongation defects of the mitotic spindle [30]. Therefore, ADD1 is essential for proper mitotic spindle assembly, while the phosphorylation of ADD1 at S726 is crucial for mitotic spindle pole integrity. However, whether ADD1 regulates meiotic spindle assembly through the same mechanism as it regulates mitotic spindle assembly remains largely unexplored.

In the present study, we surprisingly have found that ADD1 accumulated specifically at the meiotic spindle poles in mouse oocytes at the metaphase I (MI) and metaphase II (MII) stages. This observation prompted us to wonder whether ADD1 was phosphorylated at S726 during oocyte meiosis. Our results indicated that ADD1 was indeed phosphorylated at S726 throughout oocyte meiotic maturation. While S726-phosphorylated ADD1 was specifically localized to the midbody at the telophase I stage, which is different from the localization of ADD1 at this stage. Deficiency of ADD1 or TPX2 caused defective spindle assembly. Furthermore, the expression level of ADD1 and S726-phosphorylated ADD1, as well as the spatial localization of S726-phosphorylated ADD1 were regulated by TPX2. Our results suggest that ADD1 and its upstream regulator TPX2 are crucial regulators of meiotic spindle assembly in mouse oocytes.

## Results

### Expression and subcellular localization of ADD1 during mouse oocyte meiotic maturation

**Fig. 1:**
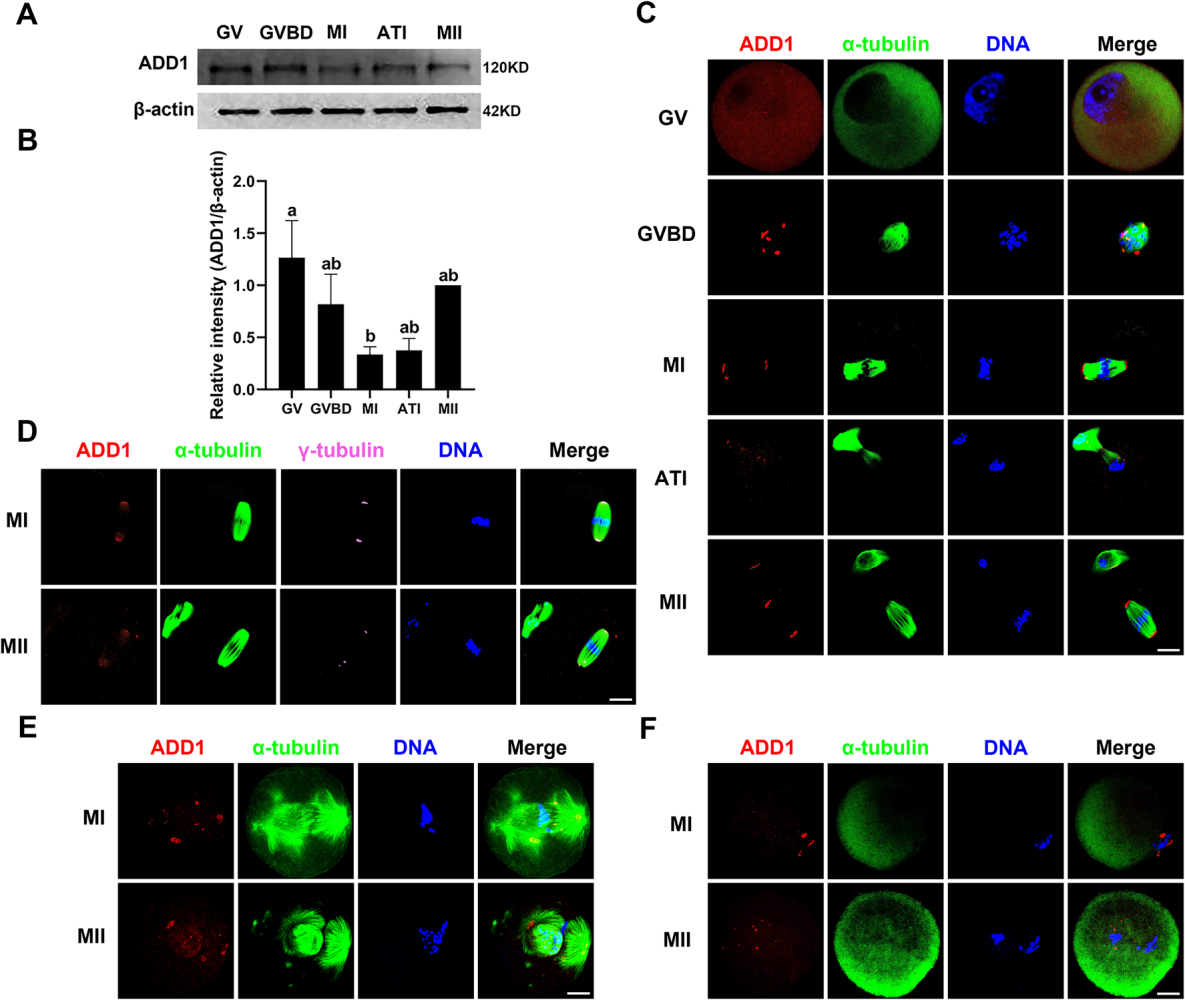
Expression and subcellular localization of ADD1 during mouse oocyte meiotic maturation. **A** The protein levels of ADD1 in oocytes at the GV, GVBD, MI, ATI, and MII stages were detected by immunoblotting. Protein loading was verified by the detection of β-actin. The molecular mass of target proteins is indicated on the right. **B** ADD1 was quantified for four independent repeats (normalized to β-actin, arbitrary units). Different lowercase letters above columns denote statistical difference at *p* < 0.05 by one-way ANOVA with the Tukey test. **C** Spatial distribution of ADD1 and spindle microtubules in oocytes at different stages. **D** Colocalization of ADD1 and γ-tubulin in MI and MII oocytes. **E** Localization of ADD1 in MI and MII oocytes exposed to taxol. **F** Distribution of ADD1 in MI and MII oocytes treated with nocodazole. Red, ADD1; green, α-tubulin; blue, DNA; pink, γ-tubulin; Merge, overlapping of red, green, blue, and pink. Bar, 20 μm

To detect the protein expression levels and dynamic subcellular localization of ADD1 in mouse oocytes at various stages of meiotic maturation, samples were collected after 0, 4, 8, 9.5, and 16 h of oocyte maturation culture, corresponding to germinal vesicle (GV), germinal vesicle breakdown (GVBD), metaphase I (MI), anaphase/telophase I (ATI), and metaphase II (MII) stages, respectively. Endogenous levels of ADD1 protein were detected by immunoblot analysis using an antibody against synthesized non-phosphopeptide around the phosphorylation site of serine 726 of ADD1 (T-P-S-P-F-L). The data demonstrated that ADD1 protein was expressed at all detected stages during mouse oocyte meiosis. Its expression level decreased to the lowest level in the MI stage, and then gradually increased with the progress of meiosis until the MII stage (Fig. 1A). Indeed, as shown in Fig. 1B, the protein level of ADD1 in MI oocytes was significantly lower than that in GV oocytes (n = 4, *p* < 0.05), but not significantly different from GVBD, ATI, and MII stage oocytes (n = 4, *p* > 0.05). To confirm the possible relationship between ADD1 and spindle apparatus, we double-stained with ADD1 and microtubule subunit α-tubulin antibodies. Immunofluorescent results demonstrated that ADD1 was uniformly and diffusely distributed throughout the cell except in the nucleoli in GV oocytes. Once the oocyte resumed meiosis and underwent GVBD, ADD1 migrated from the entire oocyte toward the perichromosomal region and accumulated around condensed chromosomes, where spindle microtubules were generated. When the cell cycle progressed to the MI stage, the chromosomes were aligned to the equatorial plate of the oocyte, and ADD1 was distributed on spindle poles with 1–3 spots clustered on each side. During the transition from the anaphase I (AI) to the telophase I (TI) stage (ATI), the homologous chromosomes were separated and segregated successively, and the aggregated ADD1 staining at the minus ends of the midzone microtubule bundle structure between segregating chromosomes was weakened to several faintly visible punctate staining. At the MII stage following the emission of the first polar body, the ADD1 signal concentrated at the spindle poles with a distribution similar to that in the MI stage (Fig. 1C). To further validate whether ADD1 functions as a microtubule minus end-binding protein or a component of the acentriolar microtubule-organizing centers (aMTOCs), we simultaneously labeled ADD1, α-tubulin, and γ-tubulin, a well-studied MTOC-specific protein that plays a critical role in microtubule nucleation and spindle formation in mammalian meiotic and mitotic cells [33], in oocytes. As shown in Fig. 1D, ADD1 colocalized with γ-tubulin at the spindle poles in MI and MII oocytes, suggesting that ADD1 may be one of the components of the oocyte microtubule organizing center to regulate spindle assembly. To illustrate the correlation between ADD1 and spindle microtubule dynamics, the effects of spindle-perturbing treatment with taxol and nocodazole on the localization of ADD1 in oocytes at MI and MII stages were investigated. After treatment of taxol, which can promote microtubule assembly and stabilize polymerized microtubules, the microtubule became excessively polymerized, and notably enlarged spindles and multiple asters were noticed in the cytoplasm. ADD1 signals were detected at the spindle poles and the minus ends of the astral microtubule fibers in the taxol-treated oocytes at both MI and MII stages (Fig. 1E). When oocytes were treated with nocodazole, a microtubule-depolymerizing agent, microtubule fibers were entirely disassembled, and no intact spindles were observed in these oocytes. The localization of ADD1 changed from being aggregated at the poles of the bipolar spindle to being dispersed around chromosomes in the nocodazole-treated oocytes at both MI and MII stages (Fig. 1F). These findings indicate that ADD1 is a component of the oocyte microtubule organizing center.

### Expression and subcellular localization of S726 phosphorylated ADD1 during mouse oocyte meiotic maturation

**Fig. 2:**
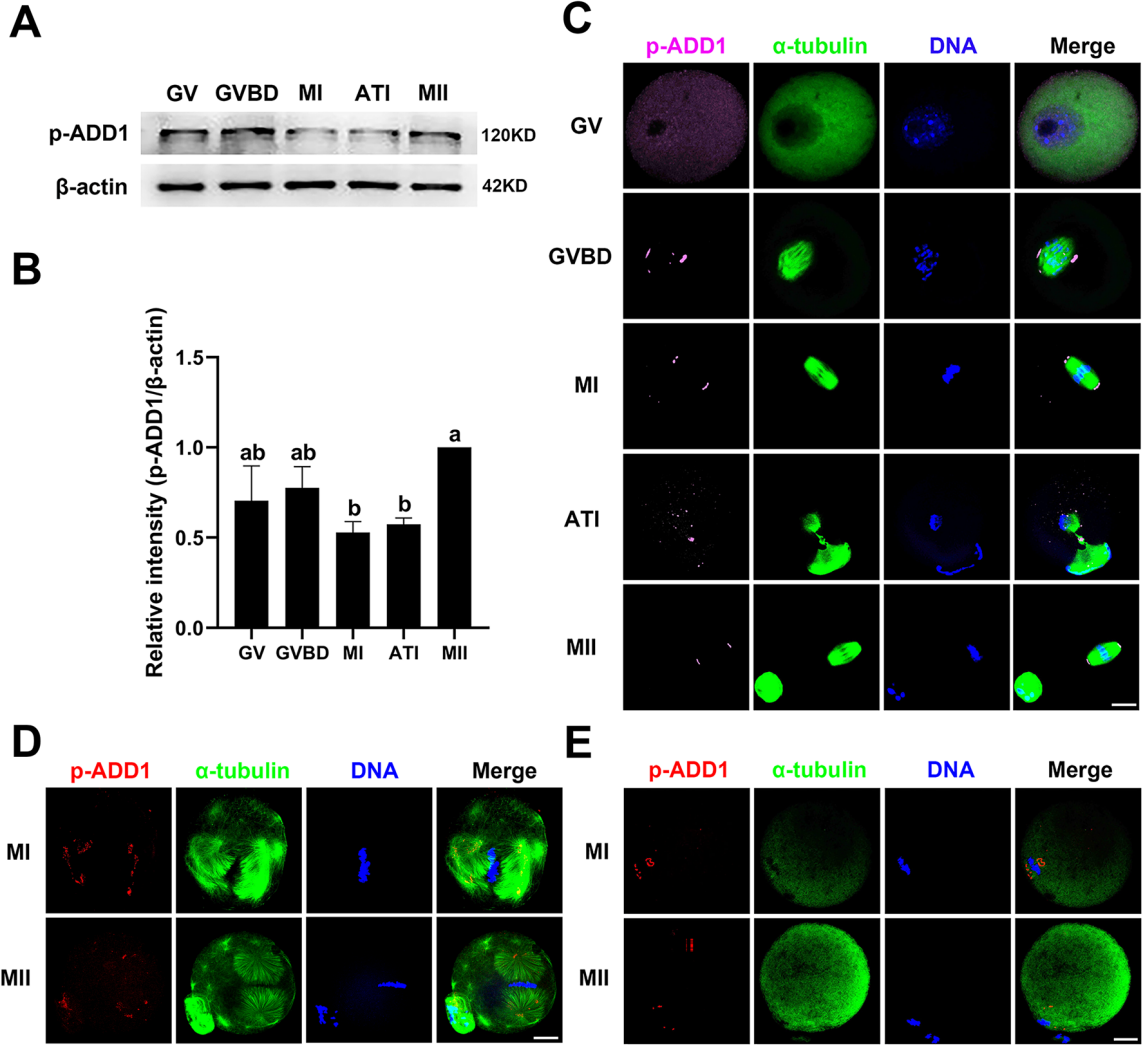
Expression and subcellular localization of p-ADD1 during mouse oocyte meiotic maturation. **A** The protein levels of p-ADD1 in oocytes at various stages were analyzed by western blot. Protein loading was verified by the detection of β-actin. The molecular mass of target proteins is indicated on the right. **B** p-ADD1 was quantified for three independent repeats (normalized to β-actin, arbitrary units). Bars that do not share the same lowercase letter are significantly different at *p* < 0.05 by one-way ANOVA with the Tukey test. **C** Immunofluorescent localization of p-ADD1 and spindle microtubules in meiotic oocytes at various stages. **D** Distribution of p-ADD1 in MI and MII oocytes treated with taxol. **E** Localization of p-ADD1 in MI and MII oocytes exposed to nocodazole. Pink or red, p-ADD1; green, α-tubulin; blue, DNA; Merge, overlapping of pink/red, green, and blue. Bar, 20 μm

Previous studies have shown that ADD1 is distributed on the mitotic spindle [30], whereas S726-phosphorylated ADD1 (p-ADD1) is specifically localized on the centrosome in somatic cells [31]. To examine whether ADD1 is phosphorylated at S726 during oocyte meiotic maturation, the protein expression levels and subcellular localization of p-ADD1 were detected by immunoblotting and immunofluorescent staining with an antibody specific for S726 phosphorylated ADD1 in mouse oocytes, respectively. ADD1 phosphorylation at S726 occurred throughout the meiotic maturation of mouse oocytes. The expression level of p-ADD1 was significantly higher in MII oocytes than in MI and ATI oocytes (n = 3, *p* < 0.05), while it was not significantly different from GV and GVBD oocytes (n = 3, *p* > 0.05). In addition, there was no significant difference in the expression of p-ADD1 between MI and ATI oocytes (n = 3, *p* > 0.05) (Fig. 2A and B). Dynamic changes in relative protein expression levels of p-ADD1 and ADD1 during oocyte meiotic maturation were not identical (Figs. 1A, B, 2A, and B), implying that the post-translational phosphorylation of ADD1 is critical for regulating its function. Immunofluorescence data indicated that the subcellular localization of p-ADD1 was the same as that of ADD1 in the GV, GVBD, MI, and MII oocytes. Strikingly, during the transition from metaphase to telophase, p-ADD1 migrated from the spindle poles to the spindle plus ends and predominantly localized in the spindle midbody, while the signal of p-ADD1 at the minus ends of the spindle was attenuated to several faintly punctate stains, in AT1 oocytes. In contrast, ADD1 demonstrated a weaker or no signal on the spindle midbody in AT1 oocytes (Figs. 1D and 2C). To further investigate the association between p-ADD1 and microtubule assembly, spindle-perturbing drugs were employed. As with the effect of taxol or nocodazole on the subcellular localization of ADD1, taxol treatment of MI and MII oocytes caused p-ADD1 to accumulate at the poles of the enlarged bipolar spindle and the minus end of astral microtubule fibers (Figs. 1F and 2D), whereas nocodazole exposure of MI and MII oocytes resulted in a dispersed distribution of p-ADD1 around the chromosomes (Figs. 1G and 2E). These results imply that p-ADD1 is involved in the microtubule nucleation in mouse oocytes.

### Disruption of ADD1 function impairs meiotic cell cycle progression in mouse oocytes

**Fig. 3:**
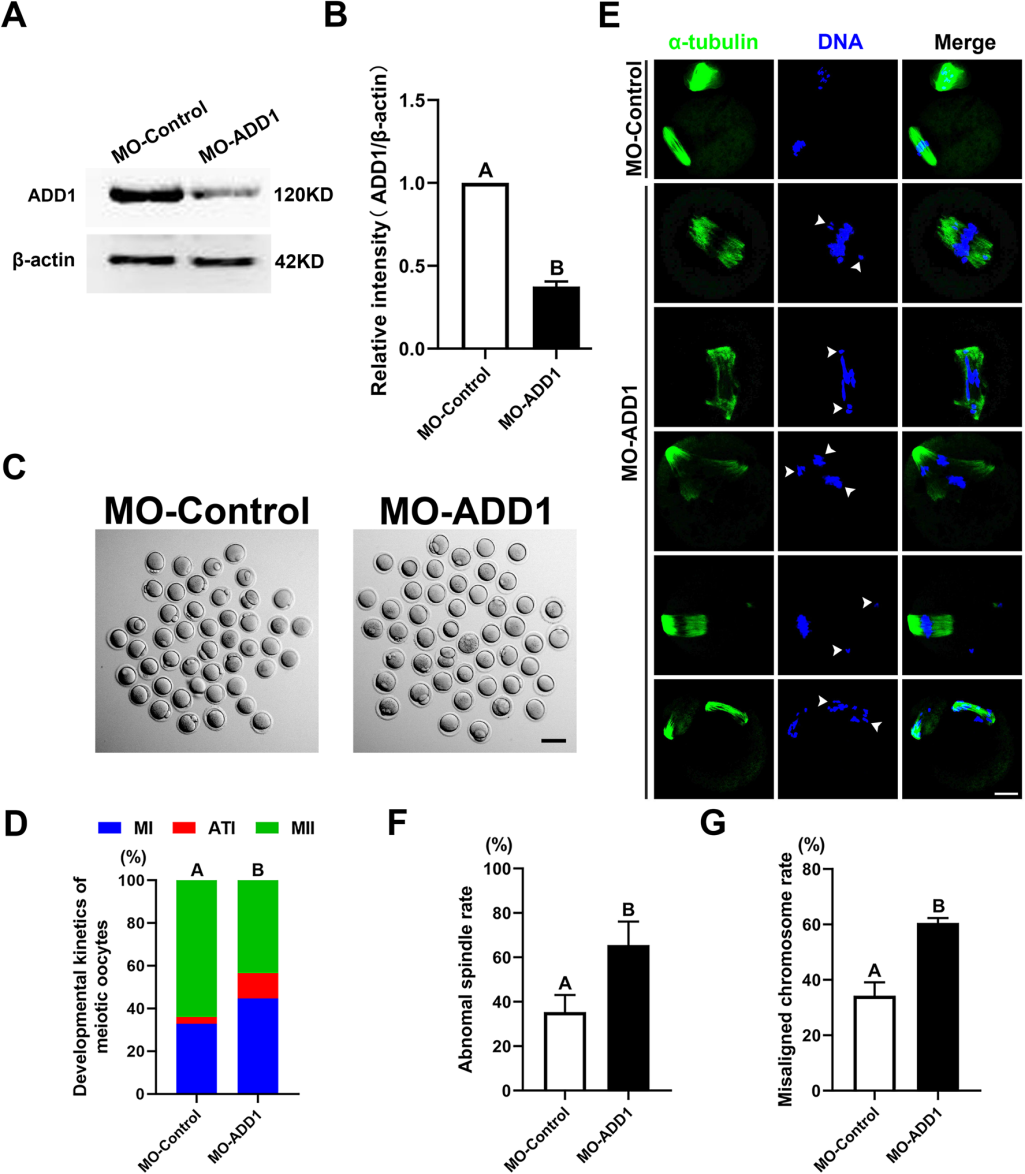
Effects of ADD1 deficiency on oocyte maturation, spindle assembly, and chromosome alignment. **A** Immunoblot probed with anti-ADD1 antibody demonstrating the depletion efficiency of ADD1-specific morpholino (MO-ADD1) in mouse oocytes. Protein loading was verified by the detection of β-actin. The molecular mass of target proteins is indicated on the right. **B** The ADD1 protein levels in control-MO- and ADD1-MO-injected oocytes were quantified for three independent repeats (normalized to β-actin, arbitrary units). In each set of experiments, the protein level of ADD1 was normalized to the value of oocytes in the control group. **C** Representative images of oocytes cultured in vitro for 16 h after control-MO or ADD1-MO treatment. Bar, 100 μm. **D** Depletion of ADD1 caused the failure of the first polar body extrusion in mouse oocytes. The rate of oocytes at the MI, AT1, and MII stages was quantified 16 h after meiotic resume in oocytes injected with control-MO and ADD1-MO, respectively. **E** Spindle morphologies and chromosome alignment 16 h after maturation culture in oocytes injected with control-MO or ADD1-MO. Green, α-tubulin; blue, DNA; Merge, overlapping of green and blue. Bar, 20 μm. **F** The rate of the oocyte with an aberrant spindle was recorded in the control-MO and ADD1-MO-injected oocytes. **G** The rate of the oocyte with misaligned chromosomes was quantified in the control and ADD1-depleted oocytes. Each column indicates the mean ± SEM of the three independent repeats in **D**, **F**, **G**. Different uppercase letters above columns denote statistical difference at *p* < 0.01 by student’s *t*-test in **B**, whereas bars that do not share the same uppercase letter are significantly different at *p* < 0.01 by the chi-square test in **D**, **F**, **G**

To clarify the function of ADD1 during mouse oocyte meiosis, a morpholino-based gene silencing approach, which provides much better specificity than RNAi, siRNA, and phosphorothioate-based oligos and greatly decreases the chance of catastrophic off-target antisense effects [34], was employed to perturb the function of ADD1. ADD1 protein expression was significantly reduced to approximately 40% in ADD1-morpholino-injected oocytes (MO-ADD1) compared to controls (MO-Control), revealing that ADD1-MO microinjection efficiently downregulated the expression of ADD1 protein in mouse oocytes (Fig. 3A and B). To investigate whether ADD1 disruption disturbs the meiotic cell cycle progression in mouse oocytes, we cultured ADD1-depleted oocytes for maturation in vitro. As shown in Fig. 3C and D, compared with the control group, ADD1 knockdown significantly reduced the proportion of oocytes with the first polar body (64.02 ± 4.81%, n = 122 vs 43.50 ± 3.28%, n = 121), increased the proportion of oocytes in AI or TI stage (3.19 ± 0.41%, n = 122 vs 11.88 ± 3.92%, n = 121).

### ADD1 deficiency leads to aberrant spindle assembly and chromosome misalignment

To investigate the effect of ADD1 on spindle assembly and chromosome arrangement, the control- and ADD1-MO-injected oocytes were collected after maturation in vitro for double staining of α-tubulin and chromosomes. As shown in Fig. 3E, a typical barrel-shaped spindle apparatus with well-aligned chromosomes on the equatorial plate was observed in most of the control oocytes. By contrast, ADD1-MO-injected oocytes exhibited a variety of abnormal spindle morphologies, including elongated spindles, spindles with widened poles, multipolar spindles, and chromosomal alignment defects manifested as randomly scattered chromosomes and chromosome bridges. The rates of distorted spindles in the MO-ADD1 group (65.58% ± 10.54%, n = 108) were significantly higher than that in the MO-Control group (35.35% ± 7.68%, n = 103) (*p* < 0.01, N = 3) (Fig. 3F). Concomitantly, an obvious increase in chromosome misalignment incidence was observed in the ADD1-MO-injected oocytes (60.57% ± 1.74%, n = 165) compared to the control-MO-injected oocytes (34.28% ± 4.79%, n = 176), (*p* < 0.01, N = 3) (Fig. 3G). Therefore, these results suggest that ADD1 regulates the progression and quality of mouse oocyte meiotic maturation by mediating bipolar spindle assembly and chromosomal alignment.

### ADD1 is necessary for the assembly of interpolar microtubules

**Fig. 4:**
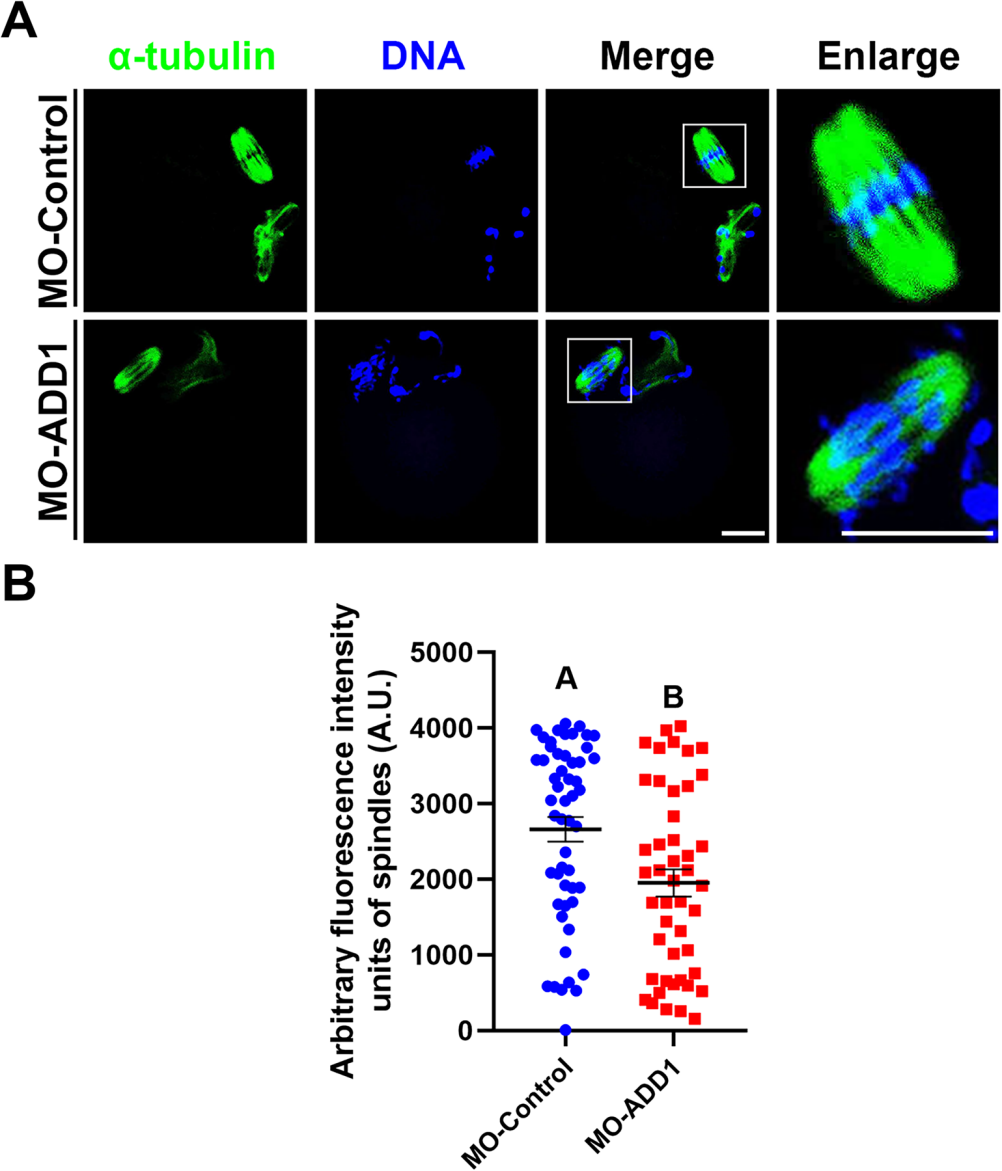
Loss of interpolar microtubules in ADD1-depleted oocytes. **A** Immunofluorescence images of the MII spindle in calcium-treated control and ADD1-depleted mouse oocytes. Green, α-tubulin; blue, DNA; Merge, overlapping of green and blue. Enlarge, an enlarged view of the image in the white boxes of the merged image. Bar, 20 μm. **B** Quantification of total fluorescence intensity (arbitrary units) of spindle microtubules in calcium-treated control and ADD1-depleted mouse oocytes at the MII stage. In each set of experiments, the fluorescence intensity of spindle microtubule staining was normalized to the value of oocytes in the control group. Bars that do not share the same uppercase letter are significantly different at *p* < 0.01 by the student’s *t*-test

To further investigate the role of ADD1 in maintaining proper morphology and function of the meiotic spindle, we examined the impact of ADD1 knockdown on the stability of K-fibers and interpolar microtubules in mouse oocytes. To assess K-fibers' stability, we treated MII oocytes, which had been injected with either control- or ADD-MO, ice-cold for 15 min to depolymerize dynamic microtubules within the meiotic spindle with preserving the stable kinetochore-bound microtubules [35, 36]. K-fibers were comparable between the control- and ADD1-MO-injected eggs (*p* > 0.05, data not shown), which implies that the connections of the k-fibers to the kinetochores and spindle poles were still intact after ADD1 deficiency by approximately 60% in mouse oocytes. To evaluate the stability of interpolar microtubules, which are the most dynamic and abundant subclass of spindle microtubules and comprise the main body of the mature spindle [37], oocytes were exposed to calcium to depolymerize fragmentary or free microtubules within the acentriolar spindle while preserving both K-fibers and stable interpolar microtubules [35, 36]. As shown in Fig. 4A and B, the fluorescence intensity of the stable interpolar microtubules in the ADD1-MO-injected oocytes (1950.16 ± 179.64 A.U., n = 46) was significantly lower than that in control-MO-injected oocytes (2660.85 ± 161.59 A.U., n = 53) (*p* < 0.01, N = 4). Overall, ADD1 is essential for interpolar microtubule stability in mouse oocyte spindles. In addition, the length and width of the spindles were marginally shorter and wider in the calcium-treated ADD1-depleted oocytes compared to their control oocytes (p > 0.05, N = 3; data not shown).

### ADD1 is required for the maintenance of euploidy in MII oocytes

**Fig. 5:**
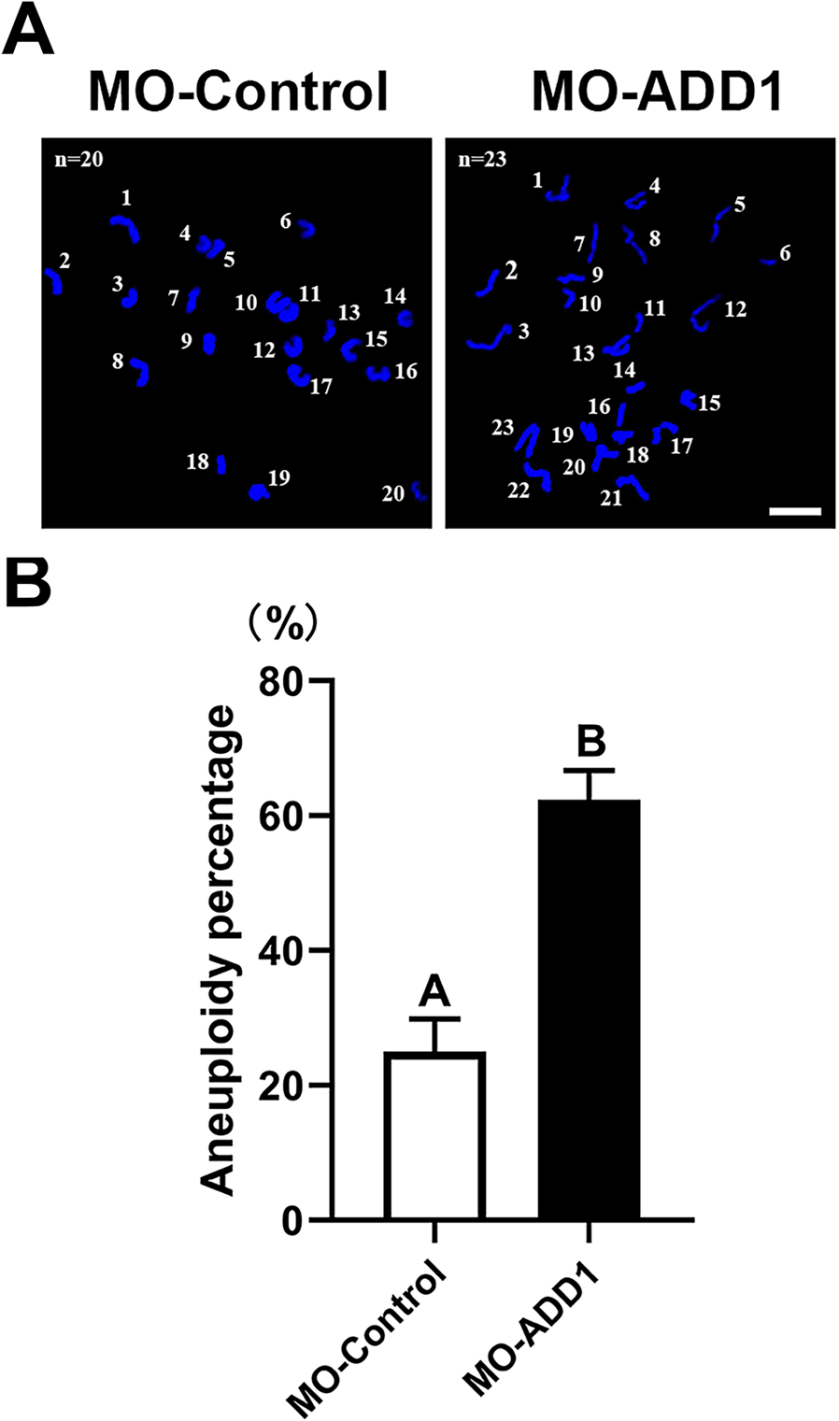
Depletion of ADD1 generates aneuploid eggs. **A** Representative images of euploid and aneuploid eggs from control and ADD1-depleted oocytes, respectively. Chromosomes were stained with DAPI. Scale bar, 5 μm. **B** The rate of aneuploid eggs was recorded in control and ADD1-depleted oocytes. Bars that do not share the same uppercase letter are significantly different at *p* < 0.01 by the chi-square test

Aberrant spindle assembly and chromosomal misalignment usually lead to aneuploidy, which causes spontaneous abortion, embryonic lethality, and ovarian teratoma [38, 39]. To elucidate whether ADD1 knockdown disrupts chromosome separation and segregation leading to aneuploidy during meiosis in mouse oocytes, we, therefore, analyzed the karyotype of MII oocytes injected with either control- or ADD1-MO by chromosome spreading. As expected, ADD1 disruption significantly increased the incidence of aneuploid with more or less 20 chromosomes, from 25.00% ± 4.81% (n = 26) in oocytes injected with control-MO to 62.39% ± 4.27% (n = 38) in the oocytes injected with ADD1-MO (*p* < 0.01, N = 3; Fig. 5A, B), suggesting that ADD1 is essential for accurate chromosome segregation during mouse oocyte meiosis.

### Expression and subcellular localization of TPX2 during mouse oocyte meiotic maturation

**Fig. 6:**
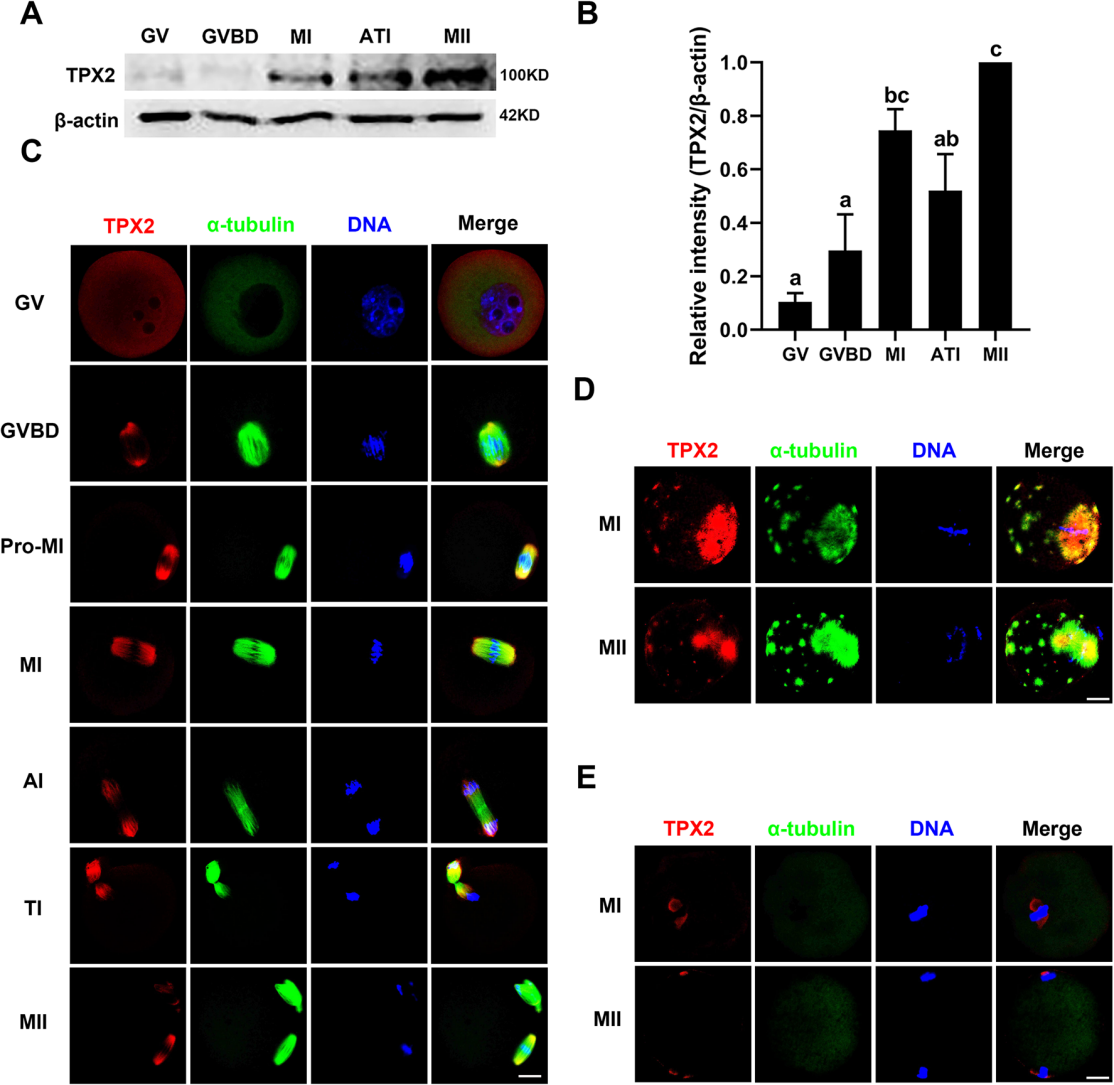
Expression and subcellular localization of TPX2 during mouse oocyte meiotic maturation. **A** The protein content of TPX2 in oocytes at the GV, GVBD, MI, ATI, and MII stages was detected by immunoblotting. Protein loading was verified by the detection of β-actin. The molecular mass of target proteins is indicated on the right. **B** TPX2 was quantified for four independent repeats (normalized to β-actin, arbitrary units). Different lowercase letters above bars indicate statistical difference at *p* < 0.05 by ANOVA with the Tukey test. **C** Immunofluorescent localization of TPX2 in meiotic oocytes at various stages. **D** Spatial distribution of TPX2 and spindle microtubules in oocytes at different meiotic maturation stages. **E** Oocytes at the MI and MII stages were treated with taxol and then double-stained for TPX2 and α-tubulin. **F** Oocytes at the MI and MII stages were exposed to nocodazole and then co-stained for p-ADD1 and α-tubulin. Red, ADD1; green, α-tubulin; blue, DNA; Merge, overlapping of red, green, and blue. Bar, 20 μm

Given that ADD1 phosphorylation at S726 is important for its interaction with TPX2, which is crucial for spindle pole integrity in somatic cells [31], this prompted us to detect the regulatory mechanisms by which ADD1 and TPX2 regulate the acentrosomal spindle assembly in mouse oocytes. To achieve this, we examined the expression and subcellular localization of TPX2 during oocyte meiotic maturation. As shown in Fig. 6A and B, the results of western blotting revealed that the overall trend of TPX2 protein level was a gradual increase with the cell cycle progression of oocyte meiosis. Quantitative analysis of western blot band density manifested that the level of TPX2 protein in MII oocytes was significantly higher than that in GV, GVBD, and ATI oocytes (N = 3, *p* < 0.05), while there was no significant difference in the level of TPX2 protein among the GV, GVBD and ATI oocytes (N = 3, *p* > 0.05). The TPX2 protein level in MI oocytes was significantly higher than that in GV and GVBD oocytes (N = 3, *p* < 0.05), whereas no significant difference was found between MI and ATI oocytes or MI and MII oocytes (N = 3, *p* > 0.05) (Fig. 6B). To further determine the relationship between the spatial distribution of TPX2 and the meiotic spindle, we double-stained oocytes at different stages with antibodies against the microtubule subunit α-tubulin and TPX2. The data of immunofluorescence demonstrated no obvious TPX2-positive signal in GV oocytes. Around GVBD, TPX2 was concentrated on newly generated spindle microtubules in perichromosomal regions. At the pro-MI or MI stage, TPX2 was localized along the spindle microtubules and was more abundant in spindle poles or minus end of spindle microtubules and less in regions close to aligned chromosomes. TPX2 staining appeared at the minus ends of the midzone microtubule bundle structure around two separate chromosomal clumps at the AI stage. It then moved to a region close to the chromosomes between two separate chromosomal clusters at the TI stage. TPX2 signaling flanked chromosomes in MII oocytes with a distribution similar to that in MI oocytes (Fig. 6C). To elucidate the correlation between TPX2 and meiotic spindle microtubule dynamics, the effects of the microtubule polymerization stabilizer taxol and the microtubule depolymerizing agent nocodazole on the subcellular localization of TPX2 in the MI and MII oocytes were investigated. TPX2 signal was detected at the minus end of both the enlarged spindle and astral microtubules in oocytes exposed to taxol (Fig. 6D). Whereas when oocytes were treated with nocodazole, the localization of TPX2 changed from being aggregated at the poles of the bipolar spindle to being randomly distributed on one or both sides of the chromosomes (Fig. 6E). These findings confirm that TPX2 is associated with microtubule nucleation and bipolar spindle assembly in mouse oocytes.

### Loss of TPX2 results in oocyte meiotic cell cycle arrest and bipolar spindle assembly failure

**Fig. 7:**
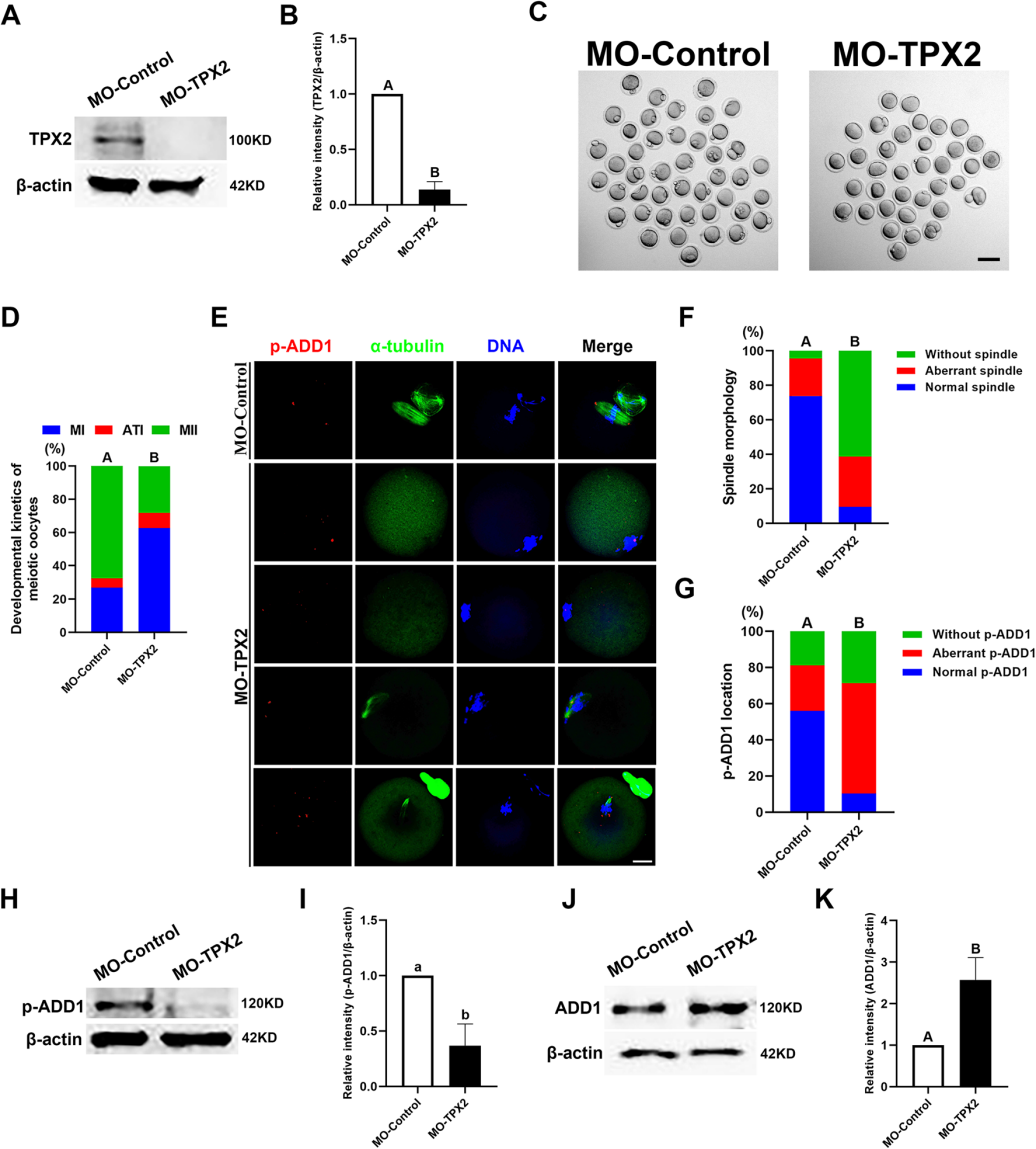
Effects of TPX2 depletion on oocyte maturation, spindle assembly, chromosome alignment, and the expression of p-ADD1 and ADD1. **A** Immunoblot probed with anti-TPX2 antibody demonstrating the depletion efficiency of TPX2-specific morpholino (MO-TPX2) in mouse oocytes. **B** TPX2 protein content in control-MO and TPX2-MO-injected oocytes was quantified for three independent repeats. **C** Representative images of oocytes cultured in vitro for 16 h after treatment with control-MO or TPX2-MO. Bar, 100 μm. **D** Depletion of TPX2 caused meiotic cell cycle arrest in MI in mouse oocytes. **E** Subcellular localization of p-ADD1, spindle morphologies, and chromosome alignment after 16 h of maturation culture in oocytes injected with control-MO or TPX2-MO. Bar, 20 μm. **F** The rate of the oocyte with a normal spindle, aberrant spindle, or no spindle was quantified in the control-MO and ADD1-MO-injected oocytes. **G** The rate of the oocyte with normal p-ADD1 localization, abnormal p-ADD1 localization, or no p-ADD1 localization was recorded in the control and ADD1 disrupted oocytes. **H** The depletion of TPX2 downregulated the phosphorylation of ADD1 at S726 in mouse oocytes. **I** The protein level of p-ADD1 in control and TPX2-depleted oocytes was quantified for three independent repeats. **J** Effect of TPX2 depletion on ADD1 protein expression in mouse oocytes. (K) ADD1 protein levels in control-MO and TPX2-MO-injected oocytes were quantified for three independent replicates. In **B**, **I**, **K**, each bar denotes the mean ± SEM of the three independent repeats, and different uppercase letters or lowercase letters above columns indicate statistical difference at *p* < 0.01 or *p* < 0.05 by student’s *t*-test, respectively. Whereas bars that do not share the same uppercase letter are significantly different at *p* < 0.01 by the chi-square test in **D**, **F**, **G**

To explore the function of TPX2 in mouse oocyte meiosis, we investigated the effects of TPX2 depletion via TPX2-targeting morpholino (MO-TPX2) on oocyte meiotic cell cycle progression, spindle assembly, and chromosomal alignment. Compared with MO-Control-injected oocytes, TPX2 protein expression was significantly reduced in MO-TPX2-injected oocytes, indicating that MO-TPX2 microinjection can effectively knockdown the expression level of TPX2 protein in mouse oocytes (Fig. 7A and B). Cell cycle assays (Fig. 7C and D) showed that 67.57 ± 5.12% (n = 99) oocytes in the MO-Control-injected group could develop to the MII stage, while in the MO-TPX2-injected group, only 28.11 ± 11.44% (n = 99) oocytes could develop to MII stage, and the remaining 62.62 ± 12.38% (n = 99) oocytes were arrested at or before MI stage. Statistical analysis demonstrated that TPX2 depletion resulted in mouse oocyte meiotic arrest in the MI stage (N = 3, *p* < 0.01). Spindle immunofluorescence assay revealed that in the MO-TPX2-injected group, 62.32 ± 14.22% (n = 86) oocytes had no or little α-tubulin signal, 29.30 ± 8.73% oocytes had monopolar aster-like spindle, and only 8.39 ± 7.20% oocytes had a normal-shaped spindle, which was significantly lower than the proportion of oocytes with a normal-shaped spindle in the MO-Control-injected group (77.07 ± 21.17%, n = 88) (N = 3, *p* < 0.01; Fig. 7E and F).

### TPX2 regulates the spindle assembly in mouse oocytes by mediating the expression and subcellular localization of S726-phosphorylated ADD1

To further reveal the regulatory relationship between ADD1 and TPX2 during spindle assembly in mouse oocytes, we examined the effect of the knockdown of ADD1 or TPX2 on each other's subcellular localization and expression levels. Our data demonstrated that the deficiency of ADD1 protein by approximately 60% had no or litter effect on the subcellular localization and the levels of TPX2 protein (data not shown). Our criteria for judging the effect of TPX2 knockdown on the localization of ADD1 or p-ADD1 were as follows: If add1 or p-ADD1 fluorescence signals existed on both sides of the chromosomes, and the sum of the fluorescence intensity on one side was comparable to the sum of the fluorescence intensity on the other side, it was considered that the localization of add1 or p-ADD1 is normal. Otherwise, add1 or p-ADD1 localization was considered abnormal if any of the conditions above were not met, or there was no visible fluorescent signal in the whole cell. Interestingly, TPX2 depletion disrupted the subcellular localization of p-ADD1 (Fig. 7E and G), reduced the expression levels of p-ADD1 (Fig. 7H, I), increased the protein content of ADD1 (Fig. 7J, K), but had no apparent effect on the subcellular localization of ADD1 (Additional file 1: Fig. S1A and S1B). These results suggest that the biological function of ADD1 requires its phosphorylation by TPX2 at S726 during mouse oocyte meiotic maturation.

## Discussion

### Progressive accumulation of TPX2 is essential for functional Meiosis I spindle assembly

Previous studies and ours demonstrate that the loss of TPX2 by RNAi or morpholino-based translation blocking results in the failure of the first meiotic spindle assembly and cell cycle arrest at or before MI in mouse oocytes [26]. Interestingly, TPX2 deficiency impairs meiotic spindle morphology and meiotic progression in a dose-dependent manner. In samples in which TPX2 siRNA-injected oocytes were maintained at the GV stage for 2 (siTPX2-2 h) or 5 (siTPX2-5 h) h prior to meiotic maturation, the treatment of siTPX2-2 h depleted about half of the TPX2 protein, whereas the siTPX2-5 h treatment almost completely depleted the protein. Compared to siTPX2-2 h treatment, siTPX2-5 h treatment displayed more perturbation in spindle assembly with only a few microtubules present around the chromosomes in most of the cases [26]. In our current research, TPX2 morpholino-injected oocytes were maintained at the GV stage for 12 h before meiotic maturation, which caused much more severe disruption in spindle structure as manifested as the complete disappearance of spindle microtubules in approximately 60% of oocytes, but compaction and aggregation of chromosomes occurred in most oocytes. The results demonstrated that microtubules first organized into a bipolar array after GVBD, and then the spindle gradually shrank, eventually forming small asters or the microtubules disappeared completely in mouse oocytes with efficient removal of endogenous TPX2 at the GV stage. In contrast, TPX2 premature overexpression at the onset of Meiosis I triggered the formation of large unorganized microtubule structures and disrupted the completion of Meiosis I in mouse oocytes [26]. Thus, the above results and ours imply that TPX2 is required for spindle maintenance to counteract overall microtubule instability, but not for initial microtubule nucleation, during the Meiosis I spindle assembly in mouse oocytes. Moreover, TPX2 mediates spindle pole organization through TACC3 phosphorylation in mouse oocytes [26], whereas our data suggest that TPX2 also controls interpolar microtubule homeostasis via ADD1 phosphorylation in mouse oocytes.

### TPX2 regulates the expression of S726-phosphorylated ADD1 in mouse oocytes

Our current results demonstrated that both ADD1 and S726-phosphorylated ADD1 exhibited dynamic changes in cell cycle-dependent expression and subcellular localization during mouse oocyte meiosis. ADD1 was specifically localized to acentriolar spindle poles or the minus end of spindle microtubules in oocytes, rather than to microfilaments in cortical areas [28, 29] or the entire spindle as in somatic cells [30]. This oocyte-specific spindle pole localization of ADD1 may be related to its phosphorylation state, such as S726 phosphorylation, which reduces the affinity of ADD1 for spectrin-F-actin complexes in vitro biochemical experiments [40], whereas S726-phosphorylated ADD1 localizes to the poles of the meiotic spindle in somatic cells [31]. In addition, phosphorylation of ADD1 at Ser12 and Ser355 allows ADD1 to interact with myosin-X and attach to the whole mitotic spindles [30]. However, the distribution of the dephosphomimetic ADD1 S726A mutant to mitotic centrosomes implies that the S726 phosphorylation of ADD1 is not required for its localization to mitotic centrosomes [31]. In the present study, although ADD1 activity was controlled by its protein levels during oocyte meiosis, the phosphorylation of ADD1 at S726 was present throughout the meiosis. The depletion of TPX2 in mouse oocytes reduced the level of S726-phosphorylated ADD1 and disrupted the normal localization of S726-phosphorylated ADD1, leading to its separation from the meiotic spindle poles or the minus end of spindle microtubules. We speculate that TPX2 may serve as a scaffold protein to recruit ADD1 to aMTOCs to regulate spindle formation and chromosome segregation.

### ADD1 mediates oocyte interpolar microtubule stability, chromosome alignment, and segregation

The typical mammalian oocyte meiotic spindle consists of two distinct subclasses of microtubules, kinetochore microtubules (K-fibers) and interpolar microtubules. Within the spindle, these subclasses of microtubules are intertwined and cooperate to ensure that the spindle is fully functional. The kinetochore microtubules link the poles of the spindle to the kinetochore of chromosomes to form K-fibers, and their main function is to separate chromosomes into daughter cells [37, 41]. Our current data demonstrated that the knockdown of ADD1 protein by approximately 60% had no obvious effect on the stability of K-fibers in mouse oocytes. However, whether the complete knockout of ADD1 protein affects the homeostasis of K-fibers in oocytes remains to be further studied. The most abundant and dynamic subclass microtubules are interpolar microtubules, which emanate from the spindle pole and extend towards the spindle equator to crosslink in an antiparallel manner with microtubules extending from opposite spindle poles. However, some interpolar microtubules are shorter and do not extend to the spindle equator [37, 41]. During anaphase, interpolar microtubules are rearranged and become the major component of the central spindle, which performs a critical role in spindle elongation to separate aligned chromosomes when microtubules grow and slide along each other [42], and induces RhoA to trigger the formation of the actomyosin contractile ring for cytokinesis [43, 44]. Accumulating evidence indicates that interpolar microtubules are essential for the establishment and maintenance of spindle bipolarization [37]. Furthermore, they regulate chromosome congression by interacting dynamically with chromosomes through the chromokinesins, such as KIF22, KIF4, and KIF15 [37, 45], or through lateral interactions with kinetochores [46–48]. Strikingly, ADD1 deficiency in the present study reduced the stability of interpolar microtubules in the meiotic spindle, resulting in chromosomal misalignment and error-prone chromosomal segregation in mouse oocytes.

## Conclusion

**Fig. 8:**
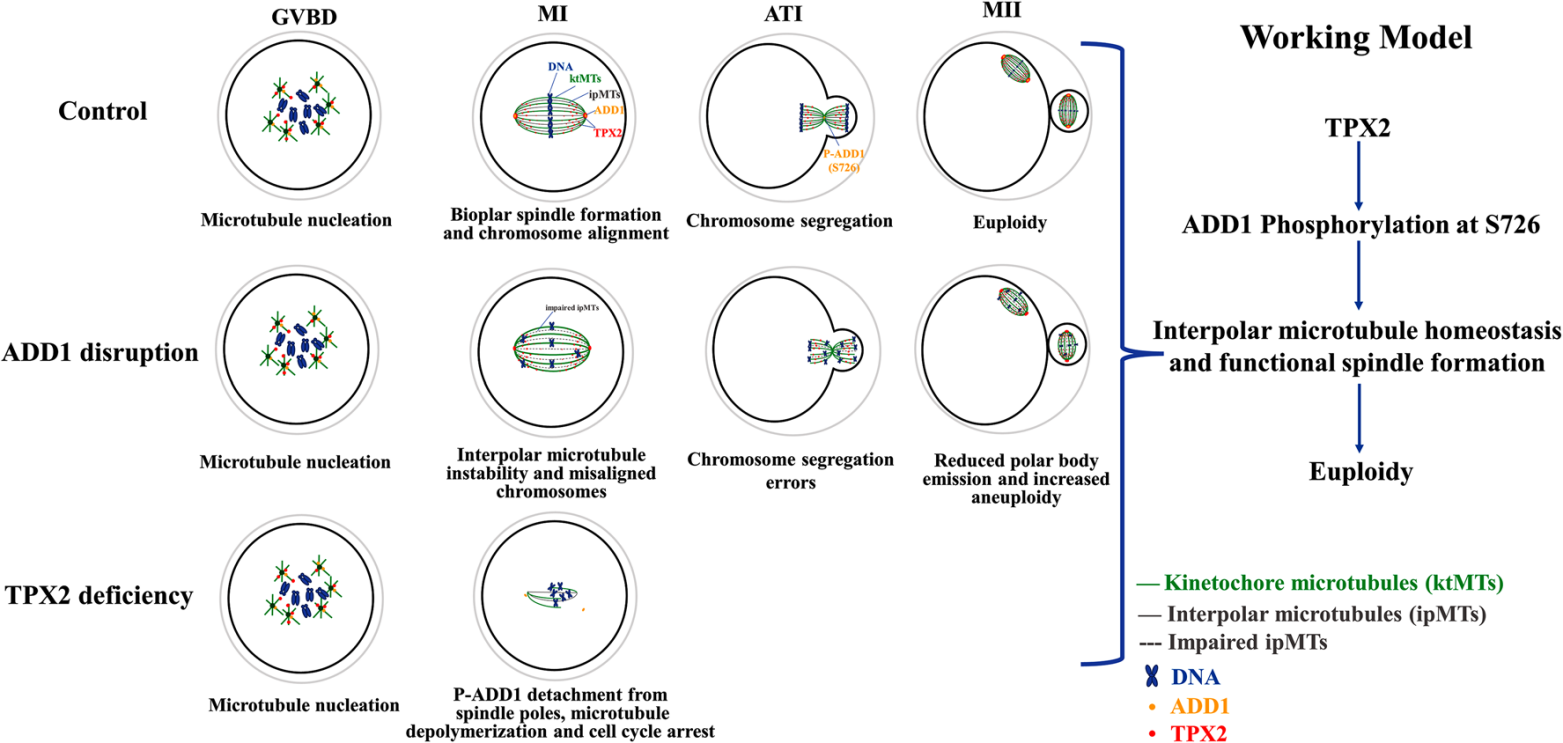
Schematic illustration of the molecular mechanism by which ADD1 regulates spindle assembly in mouse oocytes. ADD1 is phosphorylated by TPX2 and aggregates to the spindle poles to regulate the homeostasis of interpolar microtubules to ensure the formation of functional spindles and the proper segregation of chromosomes in mouse oocytes

Taken together, as illustrated in Fig. 8, our data indicate that ADD1 and TPX2 regulate mouse oocyte meiotic progression and precise chromosome segregation by regulating the homeostasis of acentriolar spindle microtubules. Disruption of ADD1 function by reducing its protein levels by 60% causes destabilization of interpolar microtubules, disrupts functional spindle assembly and precise chromosome segregation, reduces the extrusion of the first polar body, and triggers aneuploidy in MII oocytes, but has litter effect on TPX2’s function and expression in mouse oocytes. Whereas the depletion of TPX2 results in increased protein content of ADD1, reduced expression and dissociation of p-ADD1 from the spindle, spindle microtubule depolymerization, and cell cycle arrest at the MI stage. Thus, the phosphorylation of ADD1 at S726 by TPX2 is essential for functional spindle formation and precise chromosome segregation in mouse oocytes.

## Materials and methods

### Reagents and antibodies

All reagents used in this study were purchased from Sigma-Aldrich Co. (St. Louis, MO) unless otherwise noted. The rabbit polyclonal anti-ADD1 antibody (Catalog# NBP1-48,611) and the mouse monoclonal anti-TPX2 antibody (Catalog# NBP2-67,265) were purchased from Novus Biologicals. The rabbit polyclonal anti-p-ADD1 (S726) antibody (catalog# orb14892) was purchased from Biorbyt. Anti-α-tubulin-FITC (Catalog# F2168) and anti-γ-tubulin (Catalog# T6557) mouse monoclonal antibodies were obtained from Sigma-Aldrich. The mouse monoclonal anti-β-actin antibody (Catalog# ab49900) was purchased from Abcam. Alexa Fluor® 594-conjugated goat anti-rabbit IgG (H + L) (Catalog# A-11037) and Alexa Fluor® 647-conjugated goat anti-rabbit IgG (H + L) (Catalog# A-31633) were produced by Thermo Fisher Scientific Co.; TRITC conjugated goat anti-mouse IgG (H + L) (Catalog# ZF-0313) was purchased from Zhongshan Golden Bridge Biotechnology Co., LTD. Goat anti-rabbit IgG (Catalog# 1,706,515) and goat anti-mouse IgG (Catalog# 1,706,516) horseradish peroxidase (HRP)-conjugated antibodies were purchased from Bio-Rad.

### Oocyte collection and in vitro maturation

All animal protocols were approved by the Animal Ethics Committee of the Institute of Special Animal and Plant Sciences, Chinese Academy of Agricultural Sciences. Female ICR mice (4–6 weeks) were superovulated by intraperitoneal injection of 5 IU pregnant mare serum gonadotropin (PMSG) and sacrificed 46 h later. The fully-grown oocytes displaying a germinal vesicle (GV) were collected from minced ovaries in the M2 medium. Oocytes were cultured in the M16 medium under liquid paraffin oil and matured at 37 °C in an atmosphere of 5% CO_2_ and saturated humidity.

### Taxol and nocodazole treatment of oocytes

For taxol treatment, oocytes at the MI or MII stage were incubated in an M16 medium containing 10 μM taxol for 45 min. For nocodazole treatment, oocytes at the MI or MII stage were exposed to an M16 medium supplemented with 20 μg/ml nocodazole for 10 min. The oocytes exposed to DMSO in the M16 medium at the same concentration and incubation time as each treatment group were used as their controls. After treatment, oocytes were washed thoroughly and processed for immunofluorescence.

### Morpholino knockdown

The morpholino (Gene Tools, Philomath, OR, USA) sequences for ADD1 and its control were 5'-CGAGTGTCACCATTCATTGCACAAT-3' and 5'-CCTCTTACCTCAGTTACAATTTATA-3', respectively. While the morpholino sequences for TPX2 and its control were 5'-TAGTAGGGACTTGTGACATTGCCCC-3' and 5'-TACTACGGAGTTGTCACATTCCCCC-3', respectively. Fully-grown GV oocytes were collected and microinjected with 5–10 pl of 1 mM of each of the four different morpholinos in the M2 medium containing 2.5 μM milrinone at room temperature. For ADD1 knockdown, oocytes microinjected with ADD1-targeting or its control morpholino were arrested at the GV stage with 2.5 μM milrinone in an M16 medium for 24 h to facilitate the depletion of endogenous target protein under liquid paraffin oil at 37 °C in an atmosphere of 5% CO_2_ and saturated humidity. Whereas for TPX2 depletion, oocytes injected with TPX2-targeting or its control morpholino were placed in the same medium for 12 h under the same culture conditions as in ADD1 deficiency experiments. Oocytes were then collected for Western blot analysis or transferred to a milrinone-free M16 medium to resume meiosis for further experiments.

### Western blotting

For detection of ADD1, TPX2, p-ADD1, and β-actin expression, a pool of 100 oocytes at the appropriate maturation stage was lysed in 10 μl 2 × SDS loading buffer (130 mM Tris–HCl (PH 6.8), 10% β-Mercaptoethanol, 298 μM Bromophenol blue, 20% Glycerol and 5% SDS) by boiling for 10 min and stored at − 80 °C until use. The proteins in the lysed samples were separated by 10% SDS-PAGE and electrophoretically transferred onto nitrocellulose membranes at 4 °C. Membranes were blocked in TBST buffer (10 mM Tris, 150 mM NaCl, 0.1% Tween20, pH7.4) containing 5% (ADD1, p-ADD1, and β-actin) or 2% (TPX2) skimmed milk (blocking buffer) for 1.5 h at room temperature. The upper part (> 60 kDa) of the membrane was probed with either rabbit polyclonal anti-ADD1 antibody or rabbit polyclonal anti-p-ADD1 antibody, or mouse monoclonal anti-TPX2 antibody, diluted 1:1000 in blocking buffer overnight at 4 °C. The lower part (< 60 kDa) was probed with mouse monoclonal anti-β-actin antibody, diluted 1:5000 in blocking buffer overnight at 4 °C as a loading control. After three washes of 10 min each in TBST buffer, these membranes were incubated with horseradish peroxidase (HRP)-conjugated goat anti-rabbit IgG or goat anti-mouse IgG diluted 1:3000 in blocking buffer for 1 h at room temperature, respectively. The membranes were washed three times in TBST buffer and processed with the enhanced chemiluminescence (ECL) detection system (TGM) (Thermo Fisher Scientific, Cat# 32,209). All experiments were repeated at least three times starting with independently collected oocytes. Band densities were quantified by AlphaEaseFC 4.0 software.

### Immunofluorescence and confocal microscopy

For single staining of either ADD1, TPX2, or α-tubulin, oocytes were fixed in 4% paraformaldehyde in PBS for 30 min and permeabilized in PBS containing 0.5% Triton-X-100 for 30 min at room temperature. Oocytes were then blocked with 1% BSA-supplemented PBS (blocking solution) for 1 h and incubated with either rabbit polyclonal anti-ADD1 (1:100) or mouse monoclonal anti-TPX2 antibody (1:50), or mouse FITC-conjugated anti-α-tubulin antibodies (1:200) diluted with blocking solution overnight at 4 °C, respectively. Oocytes stained with anti-α-tubulin antibodies were washed three times in a washing buffer (0.1% Tween 20 and 0.01% Triton X-100 in PBS) for 5 min each and mounted on glass slides with Prolong Antifade mounting medium containing DAPI (Invitrogen, Cat# P36962). Whereas oocytes stained with anti-ADD1 or anti-TPX2 antibodies were washed three times and labeled with Alexa Fluor 594 goat anti-rabbit IgG (H + L) or Alexa Fluor 594 goat anti-mouse IgG (H + L) diluted at 1:500 with blocking solution for 1 h at room temperature, then washed 3 times and mounted on slides as described above. For double staining of ADD1/TPX2 and α-tubulin, oocytes were sequentially stained for ADD1/TPX2 and α-tubulin and mounted on slides as described above. For triple staining of γ-tubulin, ADD1, and α-tubulin, oocytes were blocked with 3% BSA-supplemented PBS (blocking solution II) for 24 h at 4 °C and incubated with mouse monoclonal anti-γ-tubulin antibody diluted 1:500 in blocking solution II overnight at 4 °C. Oocytes were washed three-time and labeled with TRITC conjugated goat anti-mouse IgG (H + L) diluted 1:100 in blocking solution II for 1 h at room temperature. After three washes, oocytes were processed according to the double staining protocol for ADD1 and α-tubulin described above. Oocytes were observed under a confocal laser scanning microscope (Nikon C2, Japan) within one week. All experiments were repeated three times starting with independently collected samples, and at least 30 oocytes were examined in each group.

### Chromosome spread

Chromosome spread of MII oocytes was performed as previously described [49] with minor changes. MII oocytes were treated with 1% sodium citrate for 15 min at room temperature. A droplet of less than 2 μl of this solution was placed on a grease-free slide with 1 oocyte. A drop of 40 μl of acetic alcohol (3 parts of absolute ethyl alcohol, 1 part of glacial acetic acid) was dropped directly above the droplet containing the oocytes. After these slides were air-dried, the chromosomes were mounted on glass slides with Prolong Antifade mounting medium containing DAPI (Invitrogen, Cat# P36962) and examined under a confocal laser-scanning microscope (Nikon C2, Japan) within one week.

### Cold- and calcium-stable assays

For the cold-resistant spindle remnants assay, oocytes at the appropriate stage were incubated in an M16 medium for 15 min at 4 ℃ and immediately fixed for routine immunofluorescence. The analysis of calcium-stable spindle microtubules was performed as previously described [36]. Briefly, oocytes at the appropriate stage were incubated in 100 mM PIPES (pH 7.0, titrated with KOH), 1 mM MgCl_2_, 0.1 mM CaCl_2,_ and 0.1% Triton X-100 at 37 °C for 15 min and immediately fixed for routine immunofluorescence.

### Statistical analysis

Statistical analyses and graphing were performed using GraphPad Prism 8 (GraphPad Software, Inc., San Diego, Ca). Each experiment was repeated at least three times, and the specific number of oocytes tested in the experiment is stated in the text. The data are shown as the mean ± SEM of independent experiments and analyzed by one-way ANOVA with the Tukey test, t-test, or Chi-square analysis. Differences at *p* < 0.05 were considered significant.

## Supplementary Information

**Additional file 1: Figure S1.** Effect of TPX2 deficiency on ADD1 subcellular localization. (A) Spatial distribution of ADD1 in control-MO and TPX2-MO-injected oocytes. Red, ADD1; green, α-tubulin; blue, DNA; Merge, overlapping of red, green, and blue. Bar, 20 μm. (B) The rate of the oocyte with normal ADD1 localization, abnormal ADD1 localization, or no ADD1 localization was recorded in the control-MO (n = 45) and TPX2-MO injected (n = 57) oocytes. Chi-square test analysis showed no significant difference between the two groups at *p* > 0.05.

## Data Availability

The original contributions presented in the study are included in the article, further inquiries can be directed to the corresponding author.

## Abbreviations

ADD1: Adducin-1
TPX2: Targeting protein for Xklp2
aMTOCs: Acentriolar microtubule organizing centers
AT1: Anaphase telophase I
CEP120/192: Centrosomal proteins 120/192
GV: Germinal vesicle
GVBD: Germinal vesicle breakdown
K-fibers: Kinetochore microtubules
KIF4/15/22: Kinesin family member 4/15/22
MI: Metaphase I
MII: Metaphase II
MYO10: Myosin X
NEDD1: Neural precursor cell expressed, developmentally down-regulated gene 1
NuMa: Nuclear mitotic apparatus protein
p-ADD1: Phosphorylated ADD1: PCMs: pericentriolar materials
PMSG: Pregnant mare serum gonadotropin
pro-MI: Pro-metaphase I
RhoA: Ras homolog family member A
SDS-PAGE: Sodium dodecyl sulfate–polyacrylamide gel electrophoresis
TBST: Tris Buffered Saline with Tween 20
