# Supplementary figures and images for: Phosphorylation of adducin-1 by TPX2 promotes interpolar microtubule homeostasis and precise chromosome segregation in mouse oocytes
Source: Cell Biosci. 2022 Dec 20;12:205. doi: 10.1186/s13578-022-00943-y (PMC9769001; doi:10.1186/s13578-022-00943-y)

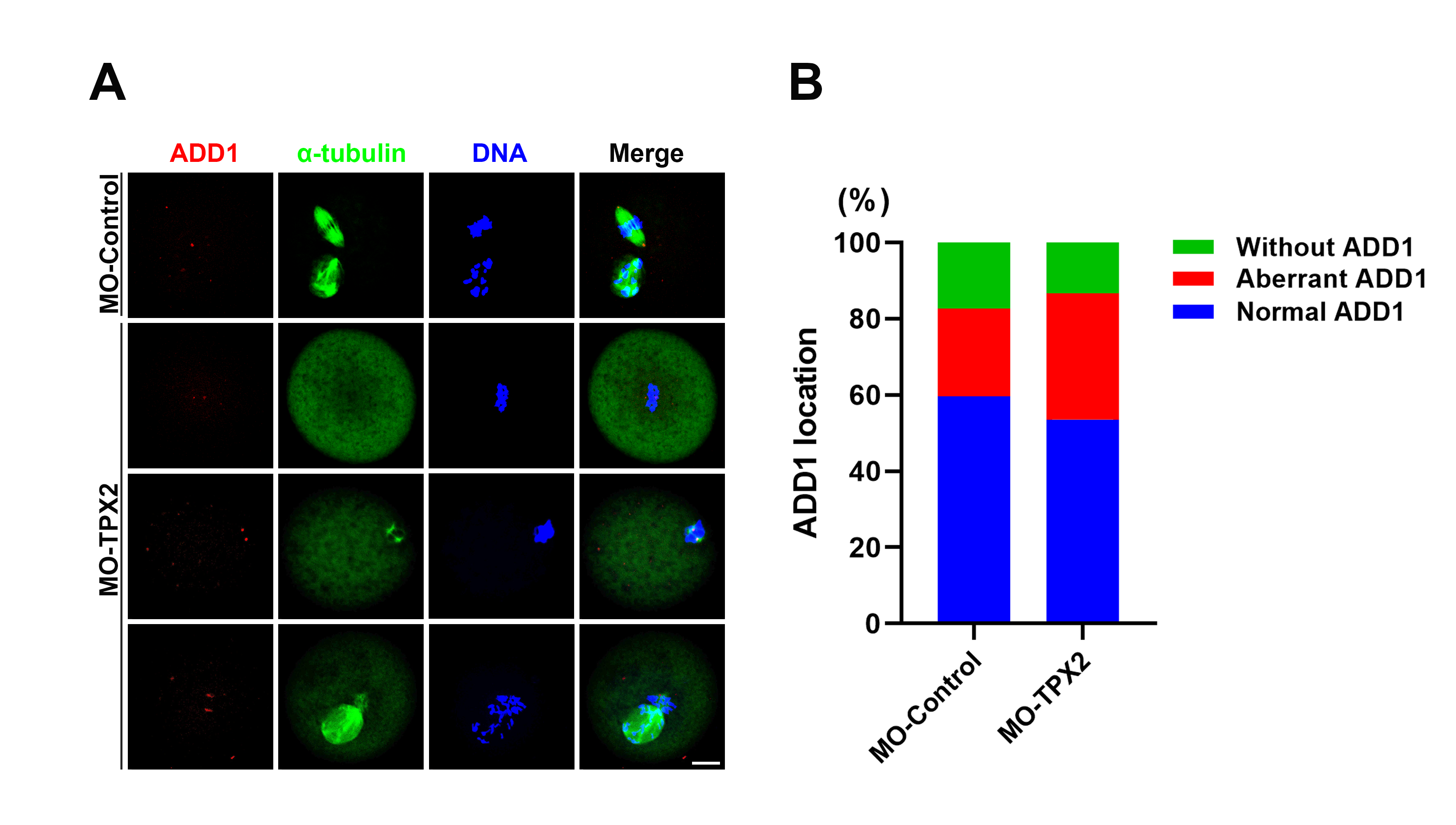

Supplement: Supplementary file 1 — Additional file 1: Figure S1. Effect of TPX2 deficiency on ADD1 subcellular localization. (A) Spatial distribution of ADD1 in control-MO and TPX2-MO-injected oocytes. Red, ADD1; green, α-tubulin; blue, DNA; Merge, overlapping of red, green, and blue. Bar, 20 μm. (B) The rate of the oocyte with normal ADD1 localization, abnormal ADD1 localization, or no ADD1 localization was recorded in the control-MO (n = 45) and TPX2-MO injected (n = 57) oocytes. Chi-square test analysis showed no significant difference between the two groups at p > 0.05. [file 13578_2022_943_MOESM1_ESM.tif]
